# Instrumental Reminiscence and Coping Strategies in Older Adults Exposed to Armed Conflict in Colombia

**DOI:** 10.1002/smi.70210

**Published:** 2026-07-19

**Authors:** Juan C. Meléndez, Tatiana Navarro, Jesus González‐Moreno, Maria Fernanda Reyes, Encarnacion Satorres, Iraida Delhom

**Affiliations:** ^1^ Department of Developmental Psychology Faculty of Psychology University of Valencia Valencia Spain; ^2^ Faculty of Education, Humanities and Arts Universidad Francisco de Paula Santander Ocaña Ocaña Norte de Santander Colombia; ^3^ Psychology Department Universidad de los Andes Bogota Colombia

**Keywords:** armed conflict, Colombia, coping strategies, instrumental reminiscence, older adults, psychosocial intervention, trauma exposure

## Abstract

Older adults exposed to prolonged sociopolitical violence often experience chronic stress that may undermine adaptive coping processes. Instrumental reminiscence, which focuses on recalling past experiences of successfully managing adversity, has been proposed as a strategy potentially associated with greater coping flexibility in later life. This study examined whether participation in an instrumental reminiscence programme was associated with changes in coping strategies among older adults registered as victims of armed conflict in Catatumbo, Colombia. A quasi‐experimental controlled design with repeated measures (pre‐intervention, post‐intervention, 2‐month follow‐up) was conducted with 64 community‐dwelling adults aged 60 years and older (intervention *n* = 37; control *n* = 27). The intervention consisted of 8 weekly 90‐min instrumental reminiscence sessions, while the comparison group received psychoeducational sessions. Coping strategies were assessed using the Coping with Stress Questionnaire. Linear mixed‐effects models were used to examine Group × Time interactions. Significant interactions were observed for problem‐solving focus, positive reappraisal, social support seeking, overt emotional expression, negative self‐focus, and avoidance (all *p* < 0.001). Compared with controls, the intervention group was characterised by higher post‐intervention scores on problem‐solving focus, positive reappraisal, social support seeking, and avoidance, and lower scores on overt emotional expression and negative self‐focus; these differences were maintained at follow‐up. No significant effects were found for religious coping. Participation in the instrumental reminiscence programme was associated with changes in multiple self‐reported coping strategies among older adults exposed to chronic conflict‐related stress.

## 1 Introduction

The identification and strengthening of effective coping strategies in response to stress arising from contexts of sociopolitical violence constitutes a priority objective of psychological intervention. In settings marked by armed conflict, recurrent threats to safety, loss of social ties, and prolonged exposure to uncertainty generate a form of chronic stress that challenges habitual coping strategies. Recent empirical evidence (Schwarzer 2024) highlights the importance of strengthening processes that facilitate more functional adaptation following traumatic experiences. Consistently, research conducted in other conflict‐affected regions, has shown that experiences of war and displacement produce profound emotional distress, accompanied by loss of identity, erosion of trust, and disruption of life meaning. However, these studies also indicate that coping responses to trauma based on meaning‐making processes and seeking social support promote psychological adaptation after continued exposure to violence (Hamadeh et al. 2023). These findings reinforce the need for contextualised psychological interventions aimed at promoting adaptive coping in populations exposed to prolonged violence.

Colombia has experienced one of the longest‐running non‐international armed conflicts in the world, characterised by a complex interaction among insurgent groups, paramilitary armed groups, and illicit economies. In rural regions of the country, this scenario has generated persistent conditions of sociopolitical violence, forced displacement, uprooting, and loss of social networks, with long‐lasting effects on mental health and coping capacity among civilians (Bernal et al. 2024). Several studies have documented high levels of psychological distress among victims of the Colombian conflict, characterised by emotional impairment and difficulties in stress regulation, along with predominantly dysfunctional coping patterns based on emotion‐focused strategies (Salas Picon et al. 2023; Ubillos‐Landa et al. 2019). These patterns, associated with continuous exposure to violence and limited community resources, may constrain long‐term psychological adaptation. In response, there has been increasing emphasis on designing culturally sensitive psychological interventions aimed at strengthening personal and emotional resources that facilitate self‐regulation and effective coping in contexts of prolonged armed conflict (Hewitt‐Ramírez et al. 2020). Although recent community‐based research in Colombia has highlighted the cumulative burden of violence, poverty, and displacement on older adults, describing faith‐based coping and emotional resignation as predominant strategies in the absence of formal psychological support (Giebel et al. 2022, 2025), there remains a need to develop structured psychological interventions aimed at strengthening individual coping resources.

Recent research has also suggested that different reminiscence functions may play an important role in psychological adaptation under adverse or traumatic conditions. Evidence from illness‐ and trauma‐related contexts indicates that self‐positive and prosocial forms of reminiscence are associated with greater post‐traumatic growth, emotional adjustment, and wellbeing, whereas self‐negative or ruminative reminiscence tends to be linked to poorer psychological outcomes and higher trauma‐related distress (Akdağ et al. 2024; Kalaycı‐Çelik and Uzer 2022). These findings suggest that reminiscence‐based processes may influence how individuals regulate emotions, construct meaning, and cope with adverse experiences. In this regard, instrumental reminiscence is not equivalent to self‐positive reminiscence as a whole. Rather, it represents a specific reminiscence function that overlaps with broader self‐positive forms of reminiscence because it reinforces perceived competence and self‐efficacy through the recall of past experiences involving the management of difficult situations, problem solving, and goal attainment.

Reminiscence is a psychological process that involves the active retrieval, review, and reinterpretation of personal past experiences to construct meaning and maintain a coherent sense of self over time, and it has been widely applied in interventions with older adults (Tam et al. 2020). Importantly, reminiscence is not a unitary construct but encompasses different functional types with distinct psychological implications (Watt and Wong 1991). From a therapeutic perspective, these functions vary in their contribution to adaptation, with instrumental reminiscence specifically referring to the recall of past experiences involving goal‐directed efforts, problem‐solving, and attempts to overcome difficulties (Wong and Watt 1991). This type of reminiscence is theoretically grounded in stress and coping frameworks, as it facilitates the identification and reactivation of previously successful coping strategies, reinforces self‐efficacy beliefs, and promotes a sense of personal competence in dealing with current stressors (Watt and Cappeliez 2000). In clinical settings, instrumental reminiscence has shown positive effects in strengthening problem‐focused coping strategies and emotional regulation, contributing to wellbeing in older adults exposed to adverse life experiences (Meléndez et al. 2015; Satorres et al. 2018). In particular, by focusing on the recall of past successful coping experiences, this approach may enhance perceived self‐efficacy and facilitate the use of more adaptive coping strategies. Consequently, this technique represents a promising tool for promoting adaptive coping processes in older adults exposed to prolonged armed conflict.

Coping constitutes an essential psychological mechanism for understanding the impact of stress and adaptive responses to adversity. According to the transactional model of stress and coping proposed by Lazarus and Folkman (1984), coping refers to the set of cognitive and behavioural efforts individuals use to manage internal or external demands perceived as threatening or exceeding their personal resources. Although strategies such as problem solving, positive reappraisal, and social support seeking are often associated with more favourable psychological adjustment, whereas negative self‐focus and dysregulated emotional expression are generally linked to greater distress, contemporary perspectives emphasise that the functional value of coping strategies depends on contextual demands and the fit between the strategy and the situation rather than on their inherent adaptiveness (Bonanno and Burton 2013; Aldao et al. 2015).

These strategies may be oriented toward problem resolution or emotional regulation, depending on available personal and contextual resources, and their adaptive value becomes particularly relevant in contexts of prolonged sociopolitical violence, such as the Colombian armed conflict, where coping processes play a central role in preserving psychological balance and functional adaptation (Schwarzer 2024; Salas Picon et al. 2023). Therefore, strengthening coping strategies represents an effective intervention pathway to promote emotional wellbeing and adaptive capacity among older adults exposed to traumatic experiences (Hewitt‐Ramírez et al. 2020).

However, evidence regarding the effects of instrumental reminiscence on coping among older adults exposed to prolonged armed conflict remains limited, particularly in Latin American conflict‐affected settings. Within this context, the present study aimed to analyse the effects of an instrumental reminiscence programme on coping strategies among older adults who are victims of the armed conflict in the Catatumbo region (Colombia). While direct evidence in conflict‐affected populations is scarce, previous studies conducted in non‐conflict contexts have shown that instrumental reminiscence can strengthen personal resources. Participation in the programme was expected to be associated with increased use of problem‐solving focus, social support seeking, and positive cognitive reappraisal. A decrease in negative self‐focus and overt emotional expression was also expected. Finally, changes in avoidance and religious coping were examined separately, given that the functional role of these strategies in contexts of sociopolitical violence may vary according to cultural, contextual, and personal factors. Overall, participation in the programme was expected to modify coping strategies, promoting more adaptive use of personal resources in response to stress derived from armed conflict.

## 2 Method

### 2.1 Study Design

A quasi‐experimental study with a comparison group and repeated measures at three time points (pre‐intervention, post‐intervention, and 2‐month follow‐up) was conducted. The study included an intervention condition based on an instrumental reminiscence programme and a psychoeducational comparison condition. Both conditions were implemented in parallel during the same time period and designed to be structurally equivalent in terms of session number, duration, group format, and level of interaction, ensuring a comparable level of participant engagement across conditions. Participants were allocated to study conditions based on their availability to attend pre‐scheduled sessions, as random assignment was not feasible in this context.

### 2.2 Participants

The sample comprised 64 older adults officially recognized as direct victims of the Colombian internal armed conflict and registered in the Single Registry of Victims (Registro Único de Víctimas, RUV) of the Unit for the Comprehensive Care and Reparation of Victims (Unidad para las Víctimas 2023). All participants resided in municipalities surrounding Ocaña, Norte de Santander (Colombia), within the Catatumbo region, historically affected by sociopolitical violence and forced displacement (Alba‐Niño 2024).

Inclusion criteria were: (a) Age 60 years or older; (b) official registration as a direct victim of the internal armed conflict in the RUV; and (c) Absence of cognitive impairment or severe disorders limiting participation. Individuals who were institutionalised or had disabling neurological conditions were excluded. Eligibility was assessed prior to study enrolment.

Initial contact with potential participants was established through psychosocial professionals and community organisations in Ocaña. Although the RUV provides large‐scale national data, disaggregated estimates for specific subpopulations (e.g., older adults in localised conflict‐affected areas) are not available for sampling purposes. Therefore, recruitment was conducted through these community‐based services, including all individuals who met the inclusion criteria and were available during the study period, without a formal sampling frame allowing estimation of the total eligible population in the area. Those who agreed to participate and met inclusion criteria were formally recruited (*N* = 71).

Due to the community‐based nature of recruitment and logistical constraints inherent to the socioterritorial context of Catatumbo, including limitations in mobility, availability, and access to meeting locations, random assignment was not operationally feasible. Participants were therefore allocated to study conditions according to their availability to attend scheduled sessions, prioritising accessibility and continuity of participation.

Of the 71 recruited participants, 39 were assigned to the intervention group and 32 to the comparison group. During the study, two participants in the intervention group (5.1%) withdrew due to medical reasons. In the comparison group, five participants (15.6%) were lost: three did not complete the post‐intervention assessment and two did not complete the follow‐up assessment. The final sample with complete data across the three assessments comprised 64 participants (37 intervention; 27 comparison).

The final sample had a mean age of 73.05 years (SD = 6.93; range = 60–89). Participants were predominantly women (76.6%, *n* = 49). Regarding marital status, 40.6% were married (*n* = 26), 40.6% widowed (*n* = 26), 12.5% separated or divorced (*n* = 8), and 6.3% single (*n* = 4). In terms of educational level, 56.2% had no formal education (28.1% illiterate and 28.1% with basic literacy), 23.5% had primary education (partial or complete), and 20.3% had secondary or higher education. Most participants belonged to low socioeconomic strata (59.4% stratum 1; 23.4% stratum 2), reflecting economic vulnerability. Regarding living arrangements, 35.9% lived alone, while the remainder lived with a partner, children, or other relatives. A total of 87.5% reported at least one chronic illness. Self‐rated health averaged 6.63 (SD = 1.82) on a scale from 2 to 10. The most frequently reported victimizing events were land abandonment or dispossession (50%), psychological injury (45.3%), loss of property (35.9%), terrorist acts or armed confrontations (35.9%), and threats (29.7%).

At baseline, the intervention group (*n* = 37) had a mean age of 72.14 years (SD = 7.27), whereas the comparison group (*n* = 27) had a mean age of 74.30 years (SD = 6.34), with no statistically significant difference (*t* (62) = 1.24, *p* = 0.220). No significant between‐group differences were observed in sex distribution (*χ* ^2^ (1) = 2.55, *p* = 0.110), marital status (*χ* ^2^ (3) = 3.05, *p* = 0.384), educational level (*χ* ^2^ (7) = 3.18, *p* = 0.868), socioeconomic level (*χ* ^2^ (2) = 1.45, *p* = 0.484), living arrangements (*χ* ^2^ (4) = 3.85, *p* = 0.427), presence of chronic illness (*χ* ^2^ (1) = 0.08, *p* = 0.774), or self‐rated health (*U* = 466.00, *p* = 0.644). Likewise, no statistically significant differences were found between groups in the distribution of victimizing events at baseline (all *p* > 0.05). Overall, the intervention and comparison groups were comparable on baseline characteristics.

All participants provided written informed consent after receiving detailed information about study objectives and procedures. The study was approved by the corresponding institutional Ethics Committee and conducted in accordance with the Declaration of Helsinki. All participants provided written informed consent.

### 2.3 Measures

A brief screening questionnaire titled Victim of the Armed Conflict in Colombia was administered to verify participants' status as victims of the internal armed conflict and to identify the type of victimising event experienced. Categories were based on the official classification of the Single Registry of Victims (Registro Único de Víctimas, RUV; Unidad para la Atención y Reparación Integral a las Víctimas, 2022) and included events such as forced land abandonment, threats, displacement, loss of property, terrorist acts, and psychological injury, among others. This instrument was used for sociodemographic purposes and to confirm eligibility criteria.

The Mini‐Mental State Examination (MMSE; Folstein et al. 1975) was used to screen for cognitive impairment. The MMSE assesses orientation, memory, attention, calculation, language, and praxis. The Colombian validated version was administered (Roselli et al. 2000). Cut‐off scores were adjusted according to educational level: 21 for individuals with six or fewer years of schooling, 24 for those with 7 to 12 years, and 27 for those with more than 12 years of education.

The Coping with Stress Questionnaire (Cuestionario de Afrontamiento del Estrés, CAE; Sandín and Chorot 2003) was used to assess coping responses to stressful situations. The CAE is a 42‐item self‐report measure rated on a five‐point Likert scale ranging from 0 (never) to 4 (almost always). Given the educational characteristics of the sample, the CAE was administered in an interviewer‐assisted format. For participants with limited literacy or no formal education, items were read aloud and clarified when needed, ensuring standardized administration across participants. The questionnaire assesses seven primary dimensions: problem‐solving focus, positive reappraisal, social support seeking, negative self‐focus, overt emotional expression, avoidance, and religious coping. These dimensions represent cognitive and behavioural strategies oriented toward problem resolution, positive reinterpretation of events, and emotional regulation through personal and social resources. Each subscale comprises six items, with scores ranging from 0 to 24, where higher scores indicate greater use of the corresponding coping strategy. It is important to note that higher scores do not uniformly reflect more adaptive coping, as some dimensions (e.g., problem‐solving focus, positive reappraisal, and social support seeking) are generally considered adaptive, whereas others (e.g., negative self‐focus and overt emotional expression) may reflect less adaptive coping patterns depending on context (Bonanno and Burton 2013; Sandín and Chorot 2003). Therefore, interpretation of the results should consider the functional nature of each coping dimension rather than assuming that higher scores are inherently beneficial. Exploratory and confirmatory factor analyses conducted by the original authors supported a seven‐factor structure and two higher‐order dimensions: problem‐focused coping (problem‐solving focus, positive reappraisal, and social support seeking) and emotion‐focused coping (negative self‐focus, emotional expression, avoidance, and religious coping). The CAE has been validated in Spanish older adult populations (Tomás et al. 2013), demonstrating adequate factorial validity and internal consistency. In the present sample, Cronbach's alpha coefficients were as follows: problem‐solving focus (0.84), positive reappraisal (0.75), social support seeking (0.90), emotional expression (0.69), negative self‐focus (0.67), avoidance (0.71), and religious coping (0.85).

### 2.4 Procedure

After confirming eligibility and obtaining informed consent, participants completed the baseline assessment, which included sociodemographic variables, victimising events, and the psychological scales used in the study. Assessments were conducted individually in face‐to‐face sessions. Participants were subsequently allocated to one of the two conditions according to their availability to attend the pre‐established time slots for each programme, prioritising accessibility and continuity of participation. Each time slot corresponded to one of the study conditions, and participants selected among available schedules without receiving information that would allow differentiation between the activities. Once assigned, they were provided with detailed information about the specific activity.

The groups then participated in their respective conditions. The experimental group attended the instrumental reminiscence program, whereas the control group participated in psychoeducational workshops focused on wellbeing promotion (sleep hygiene, nutrition, and fall prevention). Both programs were implemented in group format and delivered in parallel during the same time period, offering structured activities in each condition. Conditions were conducted separately and with different session schedules, reducing the likelihood of contamination between groups. Sessions were conducted in community facilities provided by local organizations. Post‐intervention assessment was carried out immediately after completion of the programs, and follow‐up assessment was conducted 2 months later.

#### 2.4.1 Instrumental Reminiscence Programme: Design and Theoretical Framework

The instrumental reminiscence programme was designed to strengthen coping strategies in older adults exposed to armed conflict, drawing on the transactional model of stress and coping (Lazarus and Folkman 1984) and the instrumental function of reminiscence (Wong and Watt 1991; Cappeliez and O'Rourke 2006). Within this framework, coping strategies are not considered inherently adaptive or maladaptive; rather, their functionality depends on subjective appraisal and the availability of personal and contextual resources. Accordingly, the programme aimed to enhance coping flexibility and effectiveness by promoting recognition, reinterpretation, and reuse of previously successful personal coping strategies.

The reminiscence work was structured into eight weekly group sessions of approximately 90 minutes each, led by a facilitator with prior training in psychological intervention with older adults and specific training in the instrumental reminiscence protocol used in the study. Group size ranged from 8 to 12 participants. Sessions followed a participatory and experiential format, encouraging active involvement, sharing of personal experiences, and group discussion. Each session followed three phases: (1) guided recall of autobiographical memories involving situations in which participants had faced, managed, or adapted to difficult life circumstances, (2) reflective analysis of the thoughts, behaviors, and resources involved in those experiences, and (3) transfer to the present, in which participants identified how these strategies could be applied in their current lives.

At the beginning of the program, participants were informed that sharing personal experiences was not mandatory and that they could participate at their own level of comfort. They were also encouraged to respect confidentiality within the group. Facilitators established a safe and respectful environment for group discussion.

Session content was organized according to the coping dimensions assessed by the CAE (Sandín and Chorot 2003), distributed progressively based on their functional relevance within the adaptive process. Initial sessions focused on strategies considered adaptive, such as problem‐solving focus and social support seeking. A specific session addressed “reconstruction of personal meaning,” integrating positive cognitive reappraisal and negative self‐focus, facilitating movement from self‐criticism and guilt toward a more compassionate and meaningful reinterpretation of life experiences.

Subsequent sessions addressed complementary strategies such as functional avoidance and religious coping, whose adaptive value may vary in contexts of prolonged violence. From the transactional perspective, although these strategies may not directly resolve the stressor, they may contribute to emotional regulation and meaning‐making in contexts characterized by chronic threat or loss. In the sociocultural context of Catatumbo, both were considered particularly relevant as mechanisms of self‐protection and symbolic reconstruction; therefore, their inclusion sought to promote flexible and conscious use of these resources.

A specific session focused on emotional expression, aiming to recognize its role in stress regulation and to promote more constructive and contextually appropriate forms of expression. The program concluded with an integration session to review learning and develop an individualized coping plan for everyday application.

Overall, the program sought to enhance adaptive coping strategies and coping flexibility through recognition and mobilization of prior experiences of personal efficacy. Specific objectives included: strengthening everyday problem‐solving capacity; promoting cognitive reappraisal and reconstruction of personal meaning; reinforcing social support seeking; encouraging functional use of avoidance and religious coping resources; and fostering regulated and constructive emotional expression. The structure and content of the instrumental reminiscence program sessions are summarized in Table 1.

**TABLE 1:** Structure and content of the instrumental reminiscence programme sessions.

Session/Theme	Primary CAE dimension	Instrumental objective
1. Introduction and framing		Establish a safe group environment and introduce the link between past experiences and current coping, fostering engagement and readiness for reflective work.
2. Facing past challenges	Problem‐solving focus	Recall successful problem‐solving experiences to identify transferable strategies and strengthen perceived self‐efficacy in managing current stressors.
3. Reconstructing personal meaning	Positive reappraisal/Negative self‐focus	Promote adaptive cognitive reappraisal by transforming self‐critical interpretations into more meaningful and integrative narratives, enhancing emotional regulation.
4. The strength of others	Social support	Recall experiences of giving and receiving support to reinforce help‐seeking behaviours and reactivate interpersonal coping resources.
5. Taking protective distance	Avoidance	Differentiate functional from maladaptive avoidance and promote flexible use of distancing strategies to regulate emotional overload.
6. Meaning, faith, and values	Religious coping	Strengthen meaning‐making processes by recalling experiences where beliefs and values provided psychological support in adversity.
7. Expressing emotions constructively	Overt emotional expression	Foster regulated emotional expression by reflecting on past experiences, promoting adaptive communication and emotional processing.
8. Integration and application	Integration of strategies	Consolidate coping strategies and develop an individualised plan to apply them in current and future situations.

#### 2.4.2 Comparison Condition (Control Group)

Participants assigned to the control group attended psychoeducational workshops designed to provide general health and wellbeing support, without reminiscence components. Topics included sleep hygiene, fall prevention, and healthy eating habits, adapted to the sociocultural context of Catatumbo and to participants' everyday language. Sessions were delivered over 8 weeks, with weekly meetings of approximately 90 minutes (eight sessions total), maintaining structural equivalence in duration and group format with the experimental condition. Group size was comparable to that of the intervention condition. Content was delivered primarily through oral explanations and interactive discussion. Supporting materials were designed to be accessible to participants with low literacy levels, using simple language and visual element. When written materials were provided, these were explained verbally by facilitators to ensure comprehension.

### 2.5 Data Analysis

Data were analysed using linear mixed‐effects models (LMMs), estimated separately for each subscale of the Coping with Stress Questionnaire (CAE). In each model, Time (pre‐intervention, post‐intervention, and follow‐up) was included as a within‐subject factor, and Group (control vs. intervention) as a between‐subject factor. To account for the dependency of repeated measures and interindividual variability, a random intercept for participants was specified. This random‐effects structure accounted for the correlation of repeated observations within individuals across time. Models were estimated using restricted maximum likelihood (REML). Linear mixed‐effects models were selected because they accommodate incomplete repeated‐measures data under missing‐at‐random assumptions. All analyses were conducted using R (version 4.3.2).

Fixed effects of Time, Group, and their interaction were evaluated using Type III analysis of variance with F statistics. Degrees of freedom were estimated using the Satterthwaite approximation. Post hoc comparisons were conducted based on estimated marginal means (EMMs), applying a Holm adjustment to control for Type I error in multiple comparisons. Given the number of parallel subscale analyses, results were interpreted cautiously and in relation to the overall pattern of findings across coping dimensions. Effect sizes were estimated using partial eta squared (*η* ^2^ *p*) calculated from the Type III ANOVA tables of the mixed‐effects models. Finally, descriptive statistics (mean, standard deviation, and standard error) were calculated by group, time point, and subscale to facilitate interpretation of results.

Model assumptions were examined through visual inspection of residual distributions, Q–Q plots, residuals‐versus‐fitted plots, and influential observations. Normality was assessed at the level of model residuals rather than raw CAE scores, as recommended for linear mixed‐effects models. Homoscedasticity and linearity assumptions were also evaluated based on residual plots. No major deviations from model assumptions were detected, and the original model specification was retained.

## 3 Results

Table 2 presents the means and standard deviations of the Coping with Stress Questionnaire (CAE) subscales for the intervention and control groups across the three assessment time points. At baseline, no statistically significant between‐group differences were observed on any subscale according to contrasts derived from the mixed‐effects models, indicating initial equivalence between conditions.

**TABLE 2:** Descriptive statistics (means and standard deviations) of CAE subscales by group and time point.

Subscale		Pre‐intervention mean (SD)	Post‐intervention mean (SD)	Follow‐up mean (SD)
Problem‐solving focus	Control	10.55 (5.75)	10.37 (5.37)	10.03 (5.42)
	Intervention	13.16 (5.52)	21.59 (2.32)	21.81 (2.26)
Positive reappraisal	Control	16.11 (3.40)	15.77 (3.37)	15.88 (3.46)
	Intervention	16.51 (2.61)	18.13 (1.60)	17.97 (1.67)
Social support seeking	Control	12.48 (5.89)	11.96 (5.97)	12.00 (6.09)
	Intervention	11.67 (6.23)	19.48 (4.34)	19.83 (3.98)
Overt emotional expression	Control	8.96 (3.29)	9.03 (3.39)	9.25 (3.43)
	Intervention	9.46 (5.59)	4.73 (2.20)	4.70 (2.13)
Negative self‐focus	Control	14.00 (3.07)	14.11 (2.97)	14.48 (3.12)
	Intervention	13.7 (4.07)	9.70 (3.05)	9.65 (3.32)
Avoidance	Control	14.07 (5.34)	14.11 (4.99)	14.26 (4.76)
	Intervention	13.24 (4.56)	17.08 (2.90)	17.37 (3.00)
Religious coping	Control	20.33 (5.13)	20.29 (5.05)	20.18 (4.92)
	Intervention	20.97 (2.00)	21.00 (2.83)	21.05 (2.88)

*Note:* CAE subscale scores range from 0 to 24.

Abbreviation: SD = standard deviation.

Following the intervention, statistically significant between‐group differences emerged at post‐intervention on all subscales except religious coping. Specifically, compared with the control group, the intervention group showed significantly higher scores on problem‐solving focus, positive reappraisal, social support seeking, and avoidance, and significantly lower scores on overt emotional expression and negative self‐focus. These differences were maintained at follow‐up, as intervention group scores remained stable after post‐intervention, whereas the control group showed values comparable to baseline levels.

After presenting descriptive statistics, intervention effects on coping strategies were examined using linear mixed‐effects models. Table 3 summarises the fixed effects for each CAE subscale, including main effects of Time and Group, as well as the Time × Group interaction.

**TABLE 3:** Fixed effects from the linear mixed‐effects model (LMM) across CAE subscales.

Subscale	Fixed effect	*F*	*p*	*η* ^*2*^ *p*
Problem‐solving focus	Time	24.91	< 0.001	0.29
	Group	17.34	< 0.001	0.22
	Time × group	33.51	< 0.001	0.35
Positive reappraisal	Time	78.45	< 0.001	0.56
	Group	13.72	< 0.001	0.18
	Time × group	100.71	< 0.001	0.62
Social support seeking	Time	29.13	< 0.001	0.32
	Group	2.99	0.09	0.05
	Time × group	25.86	< 0.001	0.29
Overt emotional expression	Time	27.45	< 0.001	0.31
	Group	12.12	< 0.001	0.16
	Time × group	32.10	< 0.001	0.34
Negative self‐focus	Time	77.89	< 0.001	0.56
	Group	64.13	< 0.001	0.51
	Time × group	92.10	< 0.001	0.60
Avoidance	Time	7.08	0.001	0.10
	Group	6.30	0.015	0.09
	Time × group	14.92	< 0.001	0.19
Religious coping	Time	0.02	0.98	0.00
	Group	0.62	0.44	0.01
	Time × group	0.18	0.83	0.00

Consistent with the descriptive results, significant Time × Group interactions were observed for problem‐solving focus, positive reappraisal, social support seeking, overt emotional expression, negative self‐focus, and avoidance, with partial effect sizes ranging from moderate to large (*η* ^2^ *p* = 0.19 to 0.62). No significant effects of Time, Group, or their interaction were found for religious coping.

**FIGURE 1:**
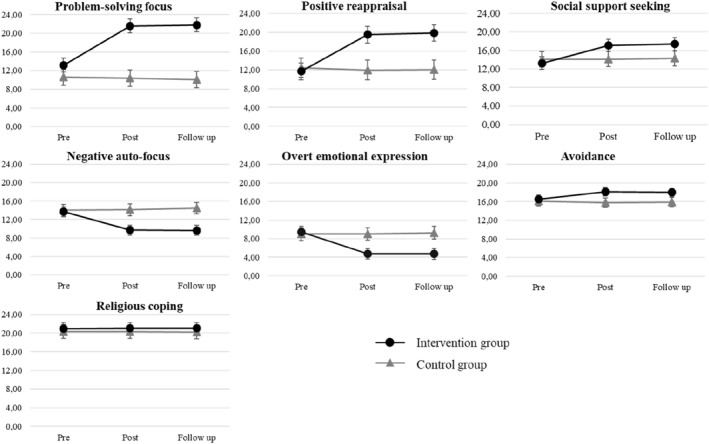
Trajectories of CAE Subscales by Group and Time. CAE subscale scores range from 0 to 24; higher scores indicate greater use of each coping strategy. Error bars represent standard errors.

To facilitate visualisation of time effects and between‐group differences identified in the linear mixed‐effects models, Figure 1 displays trajectories of CAE subscales based on estimated marginal means (EMMs) adjusted for the repeated‐measures structure of the data. The figure shows differentiated change patterns between groups across most subscales, with more pronounced changes in the intervention group after baseline that were maintained at follow‐up, whereas the control group exhibited relatively stable trajectories over time. For religious coping, both groups showed stable and comparable values across all time points.

## 4 Discussion

Overall, the intervention was associated with significant changes across multiple coping dimensions, with between‐group differences emerging after the programme and remaining stable at follow‐up. These findings support the potential of instrumental reminiscence as a psychological strategy to strengthen coping resources in older adults exposed to prolonged sociopolitical violence. These findings also suggest that instrumental reminiscence does not operate uniformly across coping dimensions, but rather differentially enhances specific strategies, particularly those linked to problem‐focused coping and cognitive reappraisal, while reducing maladaptive patterns such as negative self‐focus and dysregulated emotional expression.

Regarding problem‐oriented coping strategies, significant changes were observed in problem‐solving focus, positive reappraisal, and social support seeking in the intervention group, with improvements maintained at follow‐up. Together, these findings suggest a pattern consistent with changes in active and functional coping repertoires, particularly relevant in contexts of prolonged adversity where problem‐focused strategies have been associated with better psychological adjustment over time.

More specifically, the improvement and maintenance of problem‐solving focus may reflect a meaningful change, as this strategy is associated with greater capacity to actively manage everyday stressors and environmental demands. In trauma‐ and displacement‐exposed populations, evidence indicates that problem‐focused coping is often related to better psychological adjustment. A recent systematic review in displaced and refugee populations showed that task‐oriented or problem‐solving coping is typically associated with lower symptom severity and more adaptive functional profiles (Figueiredo and Petravičiūtė 2025). Consistent with these findings, the observed increase in problem‐solving focus may be associated with enhanced processes of self‐efficacy and active coping (Satorres et al. 2018), particularly relevant in older adults affected by sociopolitical violence, whose perceived control and agency are often eroded following chronic traumatic exposure.

The improvement observed in positive reappraisal may be indicative of changes in cognitive appraisal processes, potentially facilitating more adaptive interpretations of adverse experiences. A recent meta‐analysis reported that cognitive reappraisal is consistently associated with higher levels of resilience in the face of adversity (Stover et al. 2024). In trauma contexts, lower use of reappraisal has been linked to greater trauma symptom severity, whereas higher use is associated with reduced emotional reactivity and better functional outcomes (Ehring and Quack 2010; Tull et al. 2007). These findings suggest that enhancing the capacity to derive meaning or identify constructive aspects in difficult experiences may facilitate adaptive meaning‐making processes, central to psychological adjustment following prolonged exposure to violence.

The increase in social support seeking is particularly relevant in victimised populations, where support networks are frequently disrupted by displacement, violence, or loss of significant relationships. Previous studies have documented that armed conflicts often fracture social support systems, negatively affecting wellbeing and quality of life (Moreno‐Correa et al. 2019). Research conducted with older conflict‐affected adults in Colombia has similarly highlighted the vulnerability of community support structures in post‐conflict settings (Giebel et al. 2025). Conversely, sustained and strengthened social support has been consistently linked to improved health and quality of life in older age (Powell et al. 2020).

Taken together, these findings suggest that instrumental reminiscence, by eliciting past experiences of effective coping, may be associated with processes of self‐efficacy and resilience fostering a more adaptive reorganization of available cognitive and behavioural resources. This strengthening of problem‐oriented strategies may represent a potential mechanism underlying broader improvements in psychological adjustment and life satisfaction, as observed in recent controlled trials of instrumental reminiscence reporting significant gains in adaptation, life satisfaction, and wellbeing among older adults (Aydogdu et al. 2023).

With regard to emotion‐oriented coping strategies, exposure to war and victimising events has been associated with persistent alterations in the way individuals perceive and regulate everyday experiences. Evidence suggests that both acute and chronic stress can progressively affect cognitive, emotional, and behavioural processes, impairing cognitive flexibility and adaptive regulation, and contributing to less effective coping patterns in prolonged crisis contexts (Girotti et al. 2024). Within this framework, the intervention was associated with significant differences on negative self‐focus, overt emotional expression, and avoidance. Importantly, these coping dimensions should not be interpreted as uniformly adaptive or maladaptive, as their functional value depends on contextual and temporal factors.

A significant reduction in negative self‐focus and overt emotional expression was observed in the intervention group, a finding particularly relevant among older adults exposed to prolonged sociopolitical violence. In this context, rumination, guilt, and dysregulated emotional expression have been associated with greater psychological distress and poorer long‐term adaptation (Ubillos‐Landa et al. 2019; Salas‐Picón et al., 2023). The reduction in overt emotional expression may reflect changes in emotional regulation processes, potentially contributing to the management of distress while reducing emotional overload associated with excessive externalisation (Aldao et al. 2010). Research on emotional ageing indicates that greater emotional regulation capacity in later life is linked to better psychological adjustment and lower affective reactivity (Charles and Carstensen 2010). Similarly, the reduction in negative self‐focus suggests a decreased tendency toward self‐criticism and personal blame, potentially fostering greater self‐acceptance and a more compassionate stance toward one's experiences. Prior studies show that older adults who respond to undesired life changes with self‐directed negative affect tend to exhibit poorer psychological adjustment (Tavares et al. 2020), whereas more positive perceptions of ageing are associated with improved quality of life and emotional wellbeing (Velaithan et al. 2023).

The increase observed in avoidance warrants a cautious interpretation. Although avoidance has traditionally been associated with poorer psychological adjustment in trauma‐exposed populations, contemporary models of coping and emotion regulation suggest that the functional value of regulatory strategies depends on contextual demands and temporal dynamics, rather than reflecting uniformly adaptive or maladaptive processes (Aldao et al. 2015; Bonanno and Burton 2013). In contexts characterised by chronic and largely uncontrollable stressors, situational avoidance may serve short‐term regulatory functions by helping modulate emotional overload and preserve psychological resources. Therefore, the observed increase should not be interpreted as uniformly adaptive, but rather as potentially reflecting a context‐dependent regulatory response. Further research is needed to determine whether this pattern is associated with beneficial or maladaptive outcomes over longer periods. However, from a transactional perspective (Lazarus and Folkman 1984), avoidance is not inherently maladaptive; rather, its functionality depends on the characteristics of the stressor and the degree of controllability. In chronic and largely uncontrollable stress contexts, such as prolonged armed conflict, situational avoidance may serve short‐term regulatory functions by modulating emotional overload and preserving psychological resources. From this standpoint, the intervention may be associated with a more flexible and context‐sensitive use of avoidance as a regulatory strategy, rather than reinforcing rigid and generalised avoidance patterns. The observed increase may therefore reflect a contextual regulatory response to the evocative work inherent in instrumental reminiscence, facilitating emotional modulation without necessarily implying dysfunctional disengagement.

Trauma research indicates that individuals exposed to armed conflict may develop avoidance of both external stimuli and internal aversive experiences (thoughts, emotions, bodily sensations) as attempts at emotional regulation (Hayes et al. 1996). Although persistent avoidance patterns are associated with post‐traumatic stress symptomatology (Rothbaum et al. 1992), situational and transient avoidance may serve a protective function during certain phases of emotional processing, particularly in contexts of prolonged traumatic exposure.

Finally, religious coping remained relatively stable across time in both groups, with no significant intervention‐related changes. The consistently high levels observed across both conditions suggest that religious coping may function as a stable and widely accessible resource in this sociocultural context, rather than a strategy that is easily modified through short‐term intervention. This finding aligns with prior evidence indicating that religiosity often represents a stable and deeply rooted coping resource, particularly in conflict‐affected populations, and is less susceptible to short‐term modification through psychological intervention (Hamadeh et al. 2023). In the sociocultural context of Catatumbo, religion may function as a stable meaning framework rather than a strategy subject to immediate change, which may explain the absence of significant effects.

Taken together, findings regarding emotion‐oriented coping suggest that instrumental reminiscence may be associated with more flexible and context‐sensitive emotional regulation processes in older adults affected by sociopolitical violence. The reduction in negative self‐focus and dysregulated emotional expression, alongside a contextualised use of avoidance, points to a reorganization in how participants manage emotional distress associated with adverse experiences. This pattern supports the view that instrumental reminiscence promotes integration rather than suppression of emotional experience, facilitating acceptance, preservation of psychological resources, and adaptive adjustment in prolonged trauma contexts. This pattern, together with the reduction in overt emotional expression and the stability of religious coping, may reflect a shift toward more internally regulated and context‐sensitive coping strategies, although the adaptive value of this configuration likely depends on longer‐term outcomes.

From an applied perspective, the present findings offer relevant clinical implications for psychological interventions targeting older adults affected by sociopolitical violence. These findings support the use of instrumental reminiscence as a culturally sensitive intervention centred on personal resources and meaningful life experiences in older conflict‐affected populations. They further suggest that such interventions may be associated with the strengthening of adaptive coping strategies while modulating maladaptive emotional patterns, thereby potentially supporting more adaptive coping processes in contexts of prolonged adversity. These results align with prior research emphasising the need for interventions focused on strengthening individual psychological resources in violence‐affected populations (Hewitt‐Ramírez et al. 2020; Giebel et al. 2022). While previous initiatives have largely adopted community‐based mental health approaches, the present findings suggest that targeting individual coping processes may represent a complementary pathway for supporting coping‐related processes in older conflict‐affected adults.

Importantly, the use of an active psychoeducational comparison condition strengthens the interpretation of these findings, as it controls for non‐specific effects such as social interaction, attention, and group participation. Although nonspecific factors such as group interaction and facilitator support may have contributed to outcomes in both conditions, the structural equivalence of the comparison condition strengthens the interpretation that observed differences were associated with the specific reminiscence‐based components of the intervention.

Several limitations should be acknowledged. First, the non‐randomized allocation of participants based on availability may have introduced selection bias and limits causal inference, despite baseline equivalence between groups. Although no initial differences were observed in sociodemographic variables or coping measures, unmeasured factors may have contributed to the observed changes. Second, the sample size, while adequate for the analyses conducted, limits generalisability to other populations of older conflict victims. The community‐based recruitment approach, in the absence of a formal sampling frame, limits the ability to determine the representativeness of the sample relative to the broader population of older adults registered in the RUV in the study area. Third, reliance on self‐report measures may introduce social desirability or subjective interpretation biases. In addition, some CAE subscales, particularly negative self‐focus and overt emotional expression, showed relatively modest internal consistency in the present sample, which may have introduced measurement error and should be considered when interpreting findings related to these dimensions. The follow‐up period was relatively brief, and future studies should explore longer‐term stability of effects. Likewise, the absence of longer‐term clinical outcomes limits the ability to determine whether changes in avoidance are associated with improved or poorer long‐term adjustment. The absence of specific clinical or psychosocial outcome measures (e.g., PTSD symptoms, wellbeing, or functional adjustment) limits the ability to determine whether observed changes in coping strategies translated into broader psychological benefits. Future research should incorporate broader indicators of psychological wellbeing and psychosocial functioning to assess the overall impact of the intervention.

In conclusion, these findings provide preliminary evidence of an association between instrumental reminiscence and changes in self‐reported coping strategies among older adults affected by armed conflict. Although the present study does not allow conclusions regarding clinical or psychosocial benefit, the observed changes suggest that instrumental reminiscence may influence coping‐related processes relevant to adaptation in contexts of prolonged adversity. Future studies should incorporate broader indicators of psychological wellbeing, functioning, and trauma‐related symptoms, as well as randomized and larger‐scale designs.

## Ethics Statement

The study was approved by the Ethics Committee of the Universidad Francisco de Paula Santander, Ocaña (CEBI‐UFPSO #006).

## Conflicts of Interest

The authors declare no conflicts of interest.

## Data Availability

The data that support the findings of this study are available from the corresponding author upon reasonable request.
